# 2,4-Bis(2-chloro­phen­yl)-3-aza­bicyclo­[3.3.1]nonan-9-one

**DOI:** 10.1107/S160053680802268X

**Published:** 2008-07-26

**Authors:** P. Parthiban, V. Ramkumar, Min Sung Kim, Kwon Taek Lim, Yeon Tae Jeong

**Affiliations:** a Division of Image Science and Information Engineering, Pukyong National University, Busan 608 739, Republic of Korea; b Department of Chemistry, IIT Madras, Chennai, Tamilnadu, India

## Abstract

The mol­ecular structure of the title compound, C_20_H_19_Cl_2_NO, reveals chair conformations for both six-membered rings of the bicyclic system. Both 2-chloro­phenyl groups adopt equatorial dispositions with the chloro substituents oriented towards the carbonyl group; the aryl groups are orientated at an angle of 28.64 (3)° with respect to each other.

## Related literature

For related literature, see: Buxton *et al.* (1996 ▶); Jeyaraman *et al.* (1981 ▶); Zefirov *et al.* (1990 ▶); Vijayalakshmi *et al.* (2000 ▶); Web *et al.* (1967 ▶); Cremer & Pople (1975 ▶).
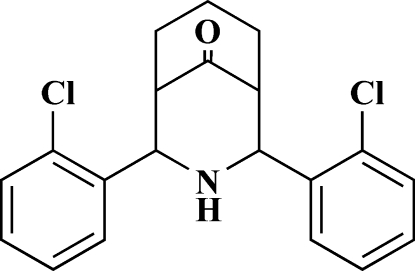


## Experimental

### 

#### Crystal data

C_20_H_19_Cl_2_NO
*M* *_r_* = 360.26
Triclinic, 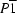

*a* = 7.7070 (15) Å
*b* = 10.680 (2) Å
*c* = 11.000 (2) Å
α = 101.78 (3)°
β = 92.82 (3)°
γ = 98.13 (3)°
*V* = 874.6 (3) Å^3^
*Z* = 2
Mo *K*α radiation
μ = 0.38 mm^−1^
*T* = 298 (2) K
0.32 × 0.25 × 0.20 mm

#### Data collection

Bruker APEXII CCD area-detector diffractometer
Absorption correction: multi-scan (*SADABS*; Bruker, 1999 ▶) *T*_min_ = 0.889, *T*_max_ = 0.928
9470 measured reflections
2949 independent reflections
2478 reflections with *I* > 2σ(*I*)
*R*_int_ = 0.026

#### Refinement

*R*[*F* ^2^ > 2σ(*F* ^2^)] = 0.087
*wR*(*F* ^2^) = 0.309
*S* = 1.19
2949 reflections
221 parameters
H atoms treated by a mixture of independent and constrained refinement
Δρ_max_ = 0.70 e Å^−3^
Δρ_min_ = −0.41 e Å^−3^

### 

Data collection: *APEX2* (Bruker–Nonius, 2004 ▶); cell refinement: *APEX2*; data reduction: *SAINT-Plus* (Bruker–Nonius, 2004 ▶); program(s) used to solve structure: *SHELXS97* (Sheldrick, 2008 ▶); program(s) used to refine structure: *SHELXL97* (Sheldrick, 2008 ▶); molecular graphics: *ORTEP-3* (Farrugia, 1997 ▶); software used to prepare material for publication: *SHELXL97*.

## Supplementary Material

Crystal structure: contains datablocks global, I. DOI: 10.1107/S160053680802268X/bx2162sup1.cif

Structure factors: contains datablocks I. DOI: 10.1107/S160053680802268X/bx2162Isup2.hkl

Additional supplementary materials:  crystallographic information; 3D view; checkCIF report

## Figures and Tables

**Fig. 1.**
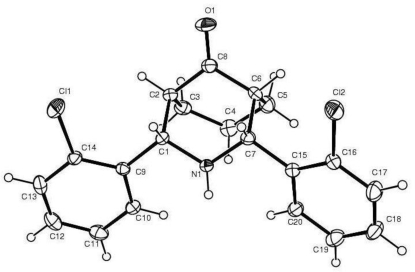
ORTEP view of the title molecule with atoms represented as 30% probability ellipsoids.

## supplementary crystallographic information

### Comment

Since the biological activities mainly depend on the stereochemistry (Jeyaraman & Avila, 1981; Buxton & Roberts, 1996) it is worthwhile to study the stereochemistry and conformation of the organic molecules. Generally, these classes of bicyclic system prefer chair-chair conformation (Zefirov & Palyulin, 1990; Vijayalakshmi *et al.*, 2000) among the three possible chair-chair, chair-boat and boat-boat conformations. However, NMR studies of this compound shows ambiguity over the conformation, due to the presence of electron withdrawing chloro substituents on *ortho* position of the either phenyl rings. Hence, we have carried out this X-ray analysis to establish the three dimensional structure.

The title compound C_20_H_19_Cl_2_NO, exists in chair-chair conformation with equatorial orientations of the *ortho* phenyl groups on both side of the secondary amino group with the torsion angles C8—C6—C7—C15 and C8—C2—C1—C9 are 178.41 (6) ° and 179.12 (6) ° respectively.

In both aryl groups, the chloro substituents point upwards *i.e.*, towards the carbonyl group and the aryl groups are orientated at an angle of 28.64 (3) ° to each other. A study of torsion angles, asymmetry parameters and least-squares plane calculation shows that the piperidine ring adopts near ideal chair conformation with a deviation of the ring atoms N1 and C8 from the C1/C2/C6/C7 plane by -0.630 (3) Å and 0.708 (3)Å respectively, Q_T_ = 0.593 (8)Å (D.Cremer & Pople, (1975)) whereas the cyclohexane ring atoms C4 and C8 deviate from the C2/C3/C5/C6 plane by -0.530 (4) Å and 0.730 (3) Å respectively (Q_T_ = 0.565 (8) Å.). Thus, indicating a deviation from the ideal chair conformation of the cyclohexane part in the title compound (Web & Becker, 1967).

### Experimental

A mixture of cyclohexanone (0.05 mol) and *ortho* chlorobenzaldehyde (0.1 mol) was added to a warm solution of ammonium acetate (0.75 mol) in 50 ml of absolute ethanol. The mixture was gently warmed on a hot plate till the yellow color formed during the mixing of the reactants and allowed to stir till the formation of the product. At the end, the pale yellow color azabicyclic ketone was separated by filtration and washed with 1:5 ethanol-ether mixture till the solid become colourless. Recrystallization of the compound from isopropyl alcohol (IPA) gave colourless crystals of 2,4-bis(2-chlorophenyl)-3-azabicyclo[3.3.1]nonan-9-one.

^1^H NMR (400 MHz, CDCl3, p.p.m.):  8.05 (dd, J = 8.0, 1.2 Hz), 7.39 (dt, J = 7.0, 1.6 Hz), 7.27 (dt, J = 7.6, 1.8 Hz), 4.85 (d, H-2a, H-4a, J = 2.4 Hz), 2.88 (m, H-7a), 2.77 (s, H-1, 5), 1.90 (d, H-8 e, J = 4.8 Hz), 1.87 (dd, H-6 e, J = 4.8, 1.6 Hz), 1.81–1.71 (m, H-8a, H-6a), 1.66 (bs, N—H), 1.41 (quintet, H-7 e).

### Refinement

Nitrogen H atoms were located in a difference Fourier map and refined isotropically. Other hydrogen atoms were fixed geometrically and allowed to ride on the parent carbon atoms,with aromatic C—H =0.93 Å, aliphatic C—H = 0.98Å and methylen C—H = 0.97 Å. The displacement parameters were set for phenyl,methylen and aliphatic H atoms at *U*_iso_(H) = 1.2*U*_eq_(C).

### Crystal data

**Table:**

C_20_H_19_Cl_2_NO	*Z* = 2
*M**_r_* = 360.26	*F*_000_ = 376
Triclinic, *P*1	*D*_x_ = 1.368 Mg m^−^^3^
Hall symbol: -P 1	Mo *K*α radiation λ = 0.71073 Å
*a* = 7.7070 (15) Å	Cell parameters from 5271 reflections
*b* = 10.680 (2) Å	θ = 2.4–28.3º
*c* = 11.000 (2) Å	µ = 0.38 mm^−^^1^
α = 101.78 (3)º	*T* = 298 (2) K
β = 92.82 (3)º	Rectangular, colourless
γ = 98.13 (3)º	0.32 × 0.25 × 0.20 mm
*V* = 874.6 (3) Å^3^	

### Data collection

**Table:**

Bruker APEXII CCD area-detector diffractometer	2949 independent reflections
Radiation source: fine-focus sealed tube	2478 reflections with *I* > 2σ(*I*)
Monochromator: graphite	*R*_int_ = 0.026
*T* = 298(2) K	θ_max_ = 25.0º
ω scans	θ_min_ = 2.7º
Absorption correction: multi-scan(SADABS; Bruker, 1999)	*h* = −8→9
*T*_min_ = 0.889, *T*_max_ = 0.928	*k* = −12→12
9470 measured reflections	*l* = −12→13

### Refinement

**Table:**

Refinement on *F*^2^	Secondary atom site location: difference Fourier map
Least-squares matrix: full	Hydrogen site location: inferred from neighbouring sites
*R*[*F*^2^ > 2σ(*F*^2^)] = 0.088	H atoms treated by a mixture of independent and constrained refinement
*wR*(*F*^2^) = 0.309	*w* = 1/[σ^2^(*F*_o_^2^) + (0.0815*P*)^2^ + 8.5055*P*] where *P* = (*F*_o_^2^ + 2*F*_c_^2^)/3
*S* = 1.19	(Δ/σ)_max_ < 0.001
2949 reflections	Δρ_max_ = 0.70 e Å^−^^3^
221 parameters	Δρ_min_ = −0.41 e Å^−^^3^
Primary atom site location: structure-invariant direct methods	Extinction correction: none

### Special details

**Table:**

Geometry. All e.s.d.'s (except the e.s.d. in the dihedral angle between two l.s. planes)are estimated using the full covariance matrix. The cell e.s.d.'s are takeninto account individually in the estimation of e.s.d.'s in distances, anglesand torsion angles; correlations between e.s.d.'s in cell parameters are onlyused when they are defined by crystal symmetry. An approximate (isotropic)treatment of cell e.s.d.'s is used for estimating e.s.d.'s involving l.s. planes.
Refinement. Refinement of *F*^2^ against ALL reflections. The weighted *R*-factor *wR* and goodness of fit *S* are based on *F*^2^, conventional *R*-factors *R* are based on *F*, with *F* set to zero for negative *F*^2^. The threshold expression of *F*^2^ > σ(*F*^2^) is used only for calculating *R*-factors(gt) *etc*. and is not relevant to the choice of reflections for refinement. *R*-factors based on *F*^2^ are statistically about twice as large as those based on *F*, and *R*- factors based on ALL data will be even larger.

### Fractional atomic coordinates and isotropic or equivalent isotropic displacement parameters (Å^2^)

**Table:**

	*x*	*y*	*z*	*U*_iso_*/*U*_eq_	
C1	0.2417 (9)	0.0189 (6)	0.1674 (6)	0.0272 (15)	
H1	0.3149	0.0273	0.0982	0.033*	
C2	0.3606 (9)	0.0002 (7)	0.2788 (7)	0.0324 (16)	
H2	0.4171	−0.0762	0.2512	0.039*	
C3	0.2618 (10)	−0.0165 (8)	0.3956 (7)	0.0377 (18)	
H3A	0.1619	−0.0848	0.3702	0.045*	
H3B	0.3398	−0.0445	0.4531	0.045*	
C4	0.1957 (11)	0.1052 (8)	0.4652 (7)	0.0425 (19)	
H4A	0.1688	0.0940	0.5479	0.051*	
H4B	0.0879	0.1153	0.4215	0.051*	
C5	0.3321 (11)	0.2301 (8)	0.4770 (7)	0.0433 (19)	
H5A	0.2734	0.3047	0.5019	0.052*	
H5B	0.4222	0.2325	0.5425	0.052*	
C6	0.4220 (9)	0.2412 (7)	0.3553 (7)	0.0339 (17)	
H6	0.5171	0.3153	0.3738	0.041*	
C7	0.2987 (9)	0.2553 (6)	0.2452 (7)	0.0291 (15)	
H7	0.3703	0.2650	0.1756	0.035*	
C8	0.5010 (9)	0.1176 (7)	0.3144 (7)	0.0329 (16)	
C9	0.0965 (9)	−0.0967 (6)	0.1234 (6)	0.0256 (14)	
C10	−0.0743 (10)	−0.0946 (8)	0.1630 (7)	0.0360 (17)	
H10	−0.0997	−0.0198	0.2142	0.043*	
C11	−0.2061 (10)	−0.2016 (8)	0.1276 (8)	0.0430 (19)	
H11	−0.3183	−0.1971	0.1539	0.052*	
C12	−0.1697 (11)	−0.3152 (8)	0.0528 (8)	0.045 (2)	
H12	−0.2570	−0.3873	0.0312	0.054*	
C13	−0.0067 (11)	−0.3211 (7)	0.0112 (7)	0.0391 (18)	
H13	0.0162	−0.3960	−0.0413	0.047*	
C14	0.1257 (9)	−0.2140 (7)	0.0478 (6)	0.0297 (15)	
C15	0.2078 (9)	0.3750 (7)	0.2778 (7)	0.0292 (15)	
C16	0.2911 (10)	0.4984 (7)	0.2705 (7)	0.0335 (16)	
C17	0.2106 (12)	0.6063 (8)	0.2961 (8)	0.047 (2)	
H17	0.2695	0.6865	0.2893	0.057*	
C18	0.0388 (12)	0.5946 (8)	0.3328 (9)	0.049 (2)	
H18	−0.0172	0.6670	0.3512	0.058*	
C19	−0.0466 (11)	0.4748 (8)	0.3413 (8)	0.046 (2)	
H19	−0.1608	0.4666	0.3656	0.055*	
C20	0.0360 (10)	0.3662 (7)	0.3141 (7)	0.0347 (17)	
H20	−0.0241	0.2861	0.3202	0.042*	
Cl1	0.3337 (3)	−0.2278 (2)	−0.0080 (2)	0.0567 (7)	
Cl2	0.5064 (3)	0.5208 (2)	0.2224 (2)	0.0501 (7)	
N1	0.1657 (8)	0.1389 (5)	0.2038 (6)	0.0290 (13)	
O1	0.6563 (7)	0.1143 (6)	0.3131 (6)	0.0523 (16)	
H1A	0.096 (12)	0.146 (8)	0.149 (9)	0.05 (3)*	

### Atomic displacement parameters (Å^2^)

**Table:**

	*U*^11^	*U*^22^	*U*^33^	*U*^12^	*U*^13^	*U*^23^
C1	0.023 (3)	0.028 (3)	0.030 (4)	0.006 (3)	0.003 (3)	0.001 (3)
C2	0.027 (4)	0.031 (4)	0.038 (4)	0.008 (3)	−0.004 (3)	0.005 (3)
C3	0.036 (4)	0.041 (4)	0.039 (4)	0.005 (3)	−0.004 (3)	0.017 (3)
C4	0.045 (5)	0.053 (5)	0.032 (4)	0.010 (4)	0.008 (4)	0.011 (4)
C5	0.050 (5)	0.043 (5)	0.033 (4)	0.010 (4)	−0.004 (4)	0.001 (3)
C6	0.025 (4)	0.029 (4)	0.043 (4)	−0.002 (3)	−0.007 (3)	0.002 (3)
C7	0.025 (4)	0.028 (3)	0.035 (4)	0.004 (3)	0.004 (3)	0.008 (3)
C8	0.028 (4)	0.036 (4)	0.035 (4)	0.006 (3)	0.000 (3)	0.008 (3)
C9	0.024 (3)	0.030 (3)	0.023 (3)	0.004 (3)	0.001 (3)	0.006 (3)
C10	0.029 (4)	0.039 (4)	0.038 (4)	0.010 (3)	0.005 (3)	0.000 (3)
C11	0.027 (4)	0.056 (5)	0.045 (5)	0.000 (4)	0.005 (3)	0.013 (4)
C12	0.048 (5)	0.039 (4)	0.042 (5)	−0.011 (4)	−0.007 (4)	0.011 (4)
C13	0.057 (5)	0.027 (4)	0.030 (4)	0.003 (3)	−0.003 (4)	0.002 (3)
C14	0.030 (4)	0.034 (4)	0.026 (3)	0.011 (3)	0.002 (3)	0.004 (3)
C15	0.028 (4)	0.029 (4)	0.031 (4)	0.005 (3)	0.000 (3)	0.006 (3)
C16	0.032 (4)	0.029 (4)	0.038 (4)	0.002 (3)	−0.001 (3)	0.005 (3)
C17	0.056 (5)	0.030 (4)	0.057 (5)	0.008 (4)	0.005 (4)	0.012 (4)
C18	0.052 (5)	0.040 (5)	0.057 (5)	0.024 (4)	0.007 (4)	0.005 (4)
C19	0.042 (5)	0.051 (5)	0.047 (5)	0.019 (4)	0.011 (4)	0.008 (4)
C20	0.032 (4)	0.033 (4)	0.040 (4)	0.008 (3)	0.004 (3)	0.009 (3)
Cl1	0.0469 (13)	0.0574 (14)	0.0617 (14)	0.0199 (10)	0.0162 (11)	−0.0077 (11)
Cl2	0.0386 (12)	0.0390 (11)	0.0702 (15)	−0.0042 (8)	0.0115 (10)	0.0113 (10)
N1	0.025 (3)	0.025 (3)	0.035 (3)	0.005 (2)	−0.005 (3)	0.004 (3)
O1	0.023 (3)	0.058 (4)	0.076 (4)	0.010 (3)	0.005 (3)	0.014 (3)

### Geometric parameters (Å, °)

**Table:**

C1—N1	1.473 (9)	C9—C14	1.408 (10)
C1—C9	1.524 (9)	C9—C10	1.408 (10)
C1—C2	1.556 (10)	C10—C11	1.393 (11)
C1—H1	0.9800	C10—H10	0.9300
C2—C8	1.507 (10)	C11—C12	1.392 (12)
C2—C3	1.554 (11)	C11—H11	0.9300
C2—H2	0.9800	C12—C13	1.364 (12)
C3—C4	1.534 (11)	C12—H12	0.9300
C3—H3A	0.9700	C13—C14	1.398 (10)
C3—H3B	0.9700	C13—H13	0.9300
C4—C5	1.555 (11)	C14—Cl1	1.759 (7)
C4—H4A	0.9700	C15—C20	1.398 (10)
C4—H4B	0.9700	C15—C16	1.401 (10)
C5—C6	1.555 (11)	C16—C17	1.372 (11)
C5—H5A	0.9700	C16—Cl2	1.766 (8)
C5—H5B	0.9700	C17—C18	1.399 (12)
C6—C8	1.525 (10)	C17—H17	0.9300
C6—C7	1.547 (10)	C18—C19	1.377 (12)
C6—H6	0.9800	C18—H18	0.9300
C7—N1	1.472 (9)	C19—C20	1.387 (11)
C7—C15	1.531 (10)	C19—H19	0.9300
C7—H7	0.9800	C20—H20	0.9300
C8—O1	1.203 (9)	N1—H1A	0.81 (9)
			
N1—C1—C9	110.5 (5)	O1—C8—C2	124.3 (7)
N1—C1—C2	109.7 (6)	O1—C8—C6	124.1 (7)
C9—C1—C2	111.4 (6)	C2—C8—C6	111.6 (6)
N1—C1—H1	108.4	C14—C9—C10	115.8 (6)
C9—C1—H1	108.4	C14—C9—C1	122.9 (6)
C2—C1—H1	108.4	C10—C9—C1	121.2 (6)
C8—C2—C3	108.7 (6)	C11—C10—C9	121.7 (7)
C8—C2—C1	107.6 (6)	C11—C10—H10	119.1
C3—C2—C1	114.4 (6)	C9—C10—H10	119.1
C8—C2—H2	108.7	C12—C11—C10	120.0 (7)
C3—C2—H2	108.7	C12—C11—H11	120.0
C1—C2—H2	108.7	C10—C11—H11	120.0
C4—C3—C2	115.2 (6)	C13—C12—C11	120.3 (7)
C4—C3—H3A	108.5	C13—C12—H12	119.9
C2—C3—H3A	108.5	C11—C12—H12	119.9
C4—C3—H3B	108.5	C12—C13—C14	119.5 (7)
C2—C3—H3B	108.5	C12—C13—H13	120.2
H3A—C3—H3B	107.5	C14—C13—H13	120.2
C3—C4—C5	112.7 (7)	C13—C14—C9	122.6 (7)
C3—C4—H4A	109.0	C13—C14—Cl1	117.5 (6)
C5—C4—H4A	109.0	C9—C14—Cl1	119.9 (5)
C3—C4—H4B	109.0	C20—C15—C16	116.6 (7)
C5—C4—H4B	109.0	C20—C15—C7	121.6 (6)
H4A—C4—H4B	107.8	C16—C15—C7	121.8 (6)
C6—C5—C4	114.2 (6)	C17—C16—C15	122.8 (7)
C6—C5—H5A	108.7	C17—C16—Cl2	116.5 (6)
C4—C5—H5A	108.7	C15—C16—Cl2	120.7 (6)
C6—C5—H5B	108.7	C16—C17—C18	119.3 (8)
C4—C5—H5B	108.7	C16—C17—H17	120.4
H5A—C5—H5B	107.6	C18—C17—H17	120.4
C8—C6—C7	107.8 (6)	C19—C18—C17	119.4 (7)
C8—C6—C5	107.0 (6)	C19—C18—H18	120.3
C7—C6—C5	115.4 (6)	C17—C18—H18	120.3
C8—C6—H6	108.9	C18—C19—C20	120.7 (8)
C7—C6—H6	108.9	C18—C19—H19	119.7
C5—C6—H6	108.9	C20—C19—H19	119.7
N1—C7—C15	109.7 (5)	C19—C20—C15	121.2 (7)
N1—C7—C6	111.0 (6)	C19—C20—H20	119.4
C15—C7—C6	112.1 (6)	C15—C20—H20	119.4
N1—C7—H7	107.9	C7—N1—C1	113.5 (5)
C15—C7—H7	107.9	C7—N1—H1A	113 (6)
C6—C7—H7	107.9	C1—N1—H1A	110 (6)
			
N1—C1—C2—C8	−58.3 (7)	C9—C10—C11—C12	1.1 (12)
C9—C1—C2—C8	179.2 (6)	C10—C11—C12—C13	−1.8 (12)
N1—C1—C2—C3	62.6 (8)	C11—C12—C13—C14	2.4 (12)
C9—C1—C2—C3	−60.0 (8)	C12—C13—C14—C9	−2.4 (11)
C8—C2—C3—C4	50.7 (8)	C12—C13—C14—Cl1	179.6 (6)
C1—C2—C3—C4	−69.5 (8)	C10—C9—C14—C13	1.7 (10)
C2—C3—C4—C5	−41.8 (9)	C1—C9—C14—C13	177.8 (7)
C3—C4—C5—C6	44.1 (9)	C10—C9—C14—Cl1	179.6 (5)
C4—C5—C6—C8	−54.4 (8)	C1—C9—C14—Cl1	−4.2 (9)
C4—C5—C6—C7	65.4 (9)	N1—C7—C15—C20	25.3 (9)
C8—C6—C7—N1	55.2 (8)	C6—C7—C15—C20	−98.5 (8)
C5—C6—C7—N1	−64.2 (8)	N1—C7—C15—C16	−153.5 (7)
C8—C6—C7—C15	178.4 (6)	C6—C7—C15—C16	82.6 (8)
C5—C6—C7—C15	59.0 (8)	C20—C15—C16—C17	−0.6 (11)
C3—C2—C8—O1	115.3 (8)	C7—C15—C16—C17	178.3 (7)
C1—C2—C8—O1	−120.3 (8)	C20—C15—C16—Cl2	−178.7 (6)
C3—C2—C8—C6	−63.3 (8)	C7—C15—C16—Cl2	0.2 (10)
C1—C2—C8—C6	61.1 (8)	C15—C16—C17—C18	0.8 (13)
C7—C6—C8—O1	121.9 (8)	Cl2—C16—C17—C18	179.0 (7)
C5—C6—C8—O1	−113.5 (8)	C16—C17—C18—C19	−0.6 (13)
C7—C6—C8—C2	−59.5 (8)	C17—C18—C19—C20	0.1 (13)
C5—C6—C8—C2	65.1 (7)	C18—C19—C20—C15	0.2 (13)
N1—C1—C9—C14	159.5 (6)	C16—C15—C20—C19	0.0 (11)
C2—C1—C9—C14	−78.4 (8)	C7—C15—C20—C19	−178.8 (7)
N1—C1—C9—C10	−24.5 (9)	C15—C7—N1—C1	178.5 (6)
C2—C1—C9—C10	97.6 (8)	C6—C7—N1—C1	−56.9 (8)
C14—C9—C10—C11	−1.0 (11)	C9—C1—N1—C7	−178.9 (6)
C1—C9—C10—C11	−177.3 (7)	C2—C1—N1—C7	58.0 (8)
